# Bis(μ-dithieno[3,2-*b*:2′,3′-*d*]thio­phene-2,6-dicarboxyl­ato-κ^2^ *O* ^2^:*O* ^6^)bis­[bis­(1,10-phenanthroline-κ^2^ *N*,*N*′)cobalt(II)] dimethyl­formamide disolvate

**DOI:** 10.1107/S1600536809011337

**Published:** 2009-04-08

**Authors:** Christopher M. MacNeill, Cynthia S. Day, Ronald E. Noftle

**Affiliations:** a Department of Chemistry, Wake Forest University, Winston-Salem, NC 27109, USA

## Abstract

The asymmetric unit of the title compound, [Co_2_(C_10_H_2_O_4_S_3_)_2_(C_12_H_8_N_2_)_4_]·2C_3_H_7_NO, contains one half of the formula unit, with the rest generated by inversion. The cobalt ion sits in a slightly distorted octa­hedral environment and is ligated to four N atoms of two 1,10-phenanthroline molecules and to two O atoms of two dithieno[3,2-*b*:2′,3′-*d*]thio­phene-2,6-dicarb­oxy­l­ate anions. The anions act as bridges between the Co^II^ centers.

## Related literature

For the synthesis of complexes with this ligand, see: Chisholm *et al.* (2008 ▶). For similar complexes, see: Xiao *et al.* (2005 ▶); Sun *et al.* (2005 ▶); Niu *et al.* (2004 ▶); Poleti *et al.* (1999 ▶).
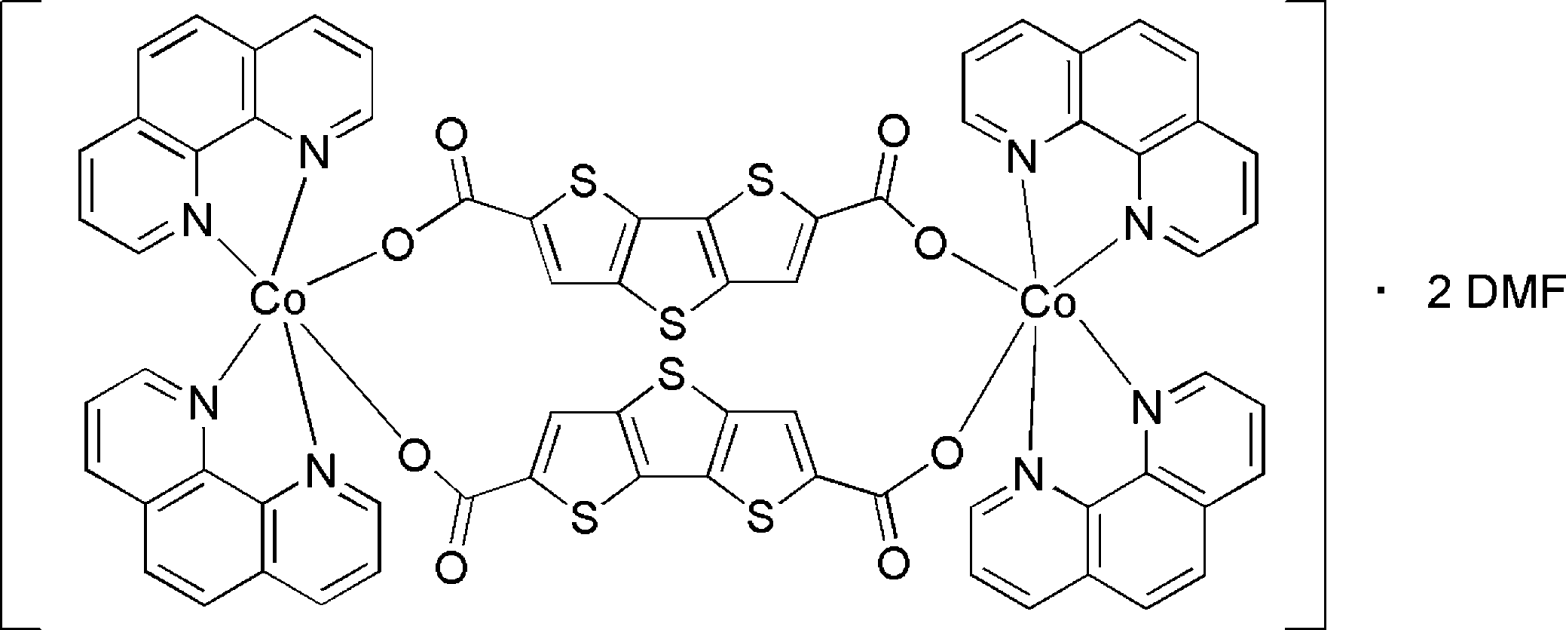


## Experimental

### 

#### Crystal data

[Co_2_(C_10_H_2_O_4_S_3_)_2_(C_12_H_8_N_2_)_4_]·2C_3_H_7_NO
*M* *_r_* = 1549.52
Triclinic, 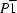

*a* = 9.884 (3) Å
*b* = 11.492 (3) Å
*c* = 15.215 (4) Å
α = 91.173 (3)°
β = 105.065 (3)°
γ = 93.057 (3)°
*V* = 1665.4 (7) Å^3^
*Z* = 1
Mo *K*α radiation
μ = 0.76 mm^−1^
*T* = 193 K
0.12 × 0.10 × 0.04 mm

#### Data collection

Bruker APEX CCD diffractometer
Absorption correction: multi-scan (*SADABS*; Sheldrick, 2008*a* ▶) *T*_min_ = 0.828, *T*_max_ = 0.970
15360 measured reflections
7532 independent reflections
5496 reflections with *I* > 2σ(*I*)
*R*_int_ = 0.045

#### Refinement

*R*[*F* ^2^ > 2σ(*F* ^2^)] = 0.062
*wR*(*F* ^2^) = 0.133
*S* = 1.06
7532 reflections
462 parameters
H-atom parameters constrained
Δρ_max_ = 0.62 e Å^−3^
Δρ_min_ = −0.44 e Å^−3^

### 

Data collection: *SMART* (Bruker, 2003 ▶); cell refinement: *SAINT* (Bruker, 2003 ▶); data reduction: *SAINT*; program(s) used to solve structure: *SHELXTL* (Sheldrick, 2008*b* ▶); program(s) used to refine structure: *SHELXTL*; molecular graphics: *SHELXTL*; software used to prepare material for publication: *SHELXTL*.

## Supplementary Material

Crystal structure: contains datablocks I, global. DOI: 10.1107/S1600536809011337/pk2151sup1.cif

Structure factors: contains datablocks I. DOI: 10.1107/S1600536809011337/pk2151Isup2.hkl

Additional supplementary materials:  crystallographic information; 3D view; checkCIF report

## Figures and Tables

**Fig. 1.**
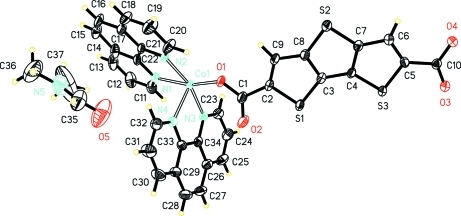
A perspective drawing of the contents of the asymmetric unit for [Co(C12H8N2)2(C10H2O4S3)]2 -2 DMF. Non-hydrogen atoms are represented by 50% probability ellipsoids.

**Fig. 2.**
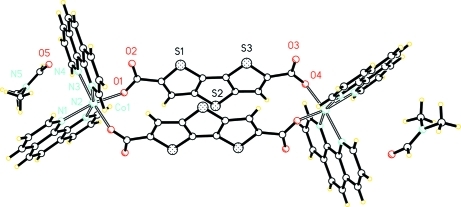
A perspective drawing of the dimeric unit for [Co(C12H8N2)2(C10H2O4S3)]2 -2 DMF. Cobalt and sulfur atoms are represented by large shaded and dotted spheres, oxygen and nitrogen atoms by medium-sized shaded spheres and carbon and hydrogen atoms by medium and small open spheres, respectively.

**Fig. 3.**
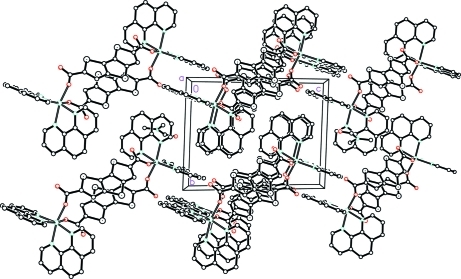
A projection down the a-axis of the unit cell in crystalline [Co(C12H8N2)2(C10H2O4S3)]2 – 2 DMF with atoms represented as in Figure 2. Hydrogen atoms have been omitted for clarity.

## supplementary crystallographic information

### Comment

We obtained the title compound during the course of our studies while forming Co^II^/1,10 phen/DTTH based coordination polymers. The asymmetric unit of the compound contains one cobalt ion along with one DTTH molecule, two 1,10-phen molecules and one lattice dimethylformamide (DMF) solvent molecule. The dimer consists of two Co(1,10-phen)_2_^2+^ cations linked by two bis-monodentate DTTH linkers. Each cobalt(II) ion is six-coordinate, forming a distorted octahedral geometry with the angles around Co1 ranging from 76.8 (1)°-104.1 (1)° and 163.9 (1)° -165.8 (1)°, respectively. Co1 is coordinated by four nitrogen atoms from two 1,10-phen moieties and two oxygen atoms from two bis-monodentate DTTH molecules. The Co—N bond lengths range from 2.127 (3)–2.192 (3)Å while the Co—O bond lengths range from 2.047 (2)–2.097 (2) Å. The angle between planes formed by the two 1,10-phen rings is 76.69 (6)°.

### Experimental

The title compound was prepared using a hydrothermal method. A mixture of cobalt nitrate pentahydrate (1.7 mmol), dithieno[3,2 - b:2',3'-d]thiophene-2,6-dicarboxylic acid (1.8 mmol) and 1,10-phenanthroline (5 mmol) were added to a vial containing DMF (1 ml) and EtOH (0.2 ml). The vial was capped and set in an oven at 105°C for 2 d. The vial was slowly cooled to room temperature to yield pink gem-like crystals.

### Crystal data

**Table:**

[Co_2_(C_10_H_2_O_4_S_3_)_2_(C_12_H_8_N_2_)_4_]·2C_3_H_7_NO	*Z* = 1
*M**_r_* = 1549.52	*F*(000) = 794
Triclinic, *P*1	*D*_x_ = 1.545 Mg m^−^^3^
Hall symbol: -P 1	Mo *K*α radiation, λ = 0.71073 Å
*a* = 9.884 (3) Å	Cell parameters from 2172 reflections
*b* = 11.492 (3) Å	θ = 3.9–22.9°
*c* = 15.215 (4) Å	µ = 0.76 mm^−^^1^
α = 91.173 (3)°	*T* = 193 K
β = 105.065 (3)°	Gem, pink
γ = 93.057 (3)°	0.12 × 0.10 × 0.04 mm
*V* = 1665.4 (7) Å^3^	

### Data collection

**Table:**

Bruker APEX CCD diffractometer	7532 independent reflections
Radiation source: fine-focus sealed tube	5496 reflections with *I* > 2σ(*I*)
graphite	*R*_int_ = 0.045
φ and ω scans	θ_max_ = 27.5°, θ_min_ = 3.9°
Absorption correction: multi-scan (*SADABS*; Sheldrick, 2008a)	*h* = −12→12
*T*_min_ = 0.828, *T*_max_ = 0.970	*k* = −14→14
15360 measured reflections	*l* = −19→19

### Refinement

**Table:**

Refinement on *F*^2^	Primary atom site location: structure-invariant direct methods
Least-squares matrix: full	Secondary atom site location: difference Fourier map
*R*[*F*^2^ > 2σ(*F*^2^)] = 0.062	Hydrogen site location: inferred from neighbouring sites
*wR*(*F*^2^) = 0.133	H-atom parameters constrained
*S* = 1.06	*w* = 1/[σ^2^(*F*_o_^2^) + (0.0536*P*)^2^ + 0.6813*P*] where *P* = (*F*_o_^2^ + 2*F*_c_^2^)/3
7532 reflections	(Δ/σ)_max_ = 0.001
462 parameters	Δρ_max_ = 0.62 e Å^−^^3^
0 restraints	Δρ_min_ = −0.44 e Å^−^^3^

### Special details

**Table:**

Geometry. All e.s.d.'s (except the e.s.d. in the dihedral angle between two l.s. planes) are estimated using the full covariance matrix. The cell e.s.d.'s are taken into account individually in the estimation of e.s.d.'s in distances, angles and torsion angles; correlations between e.s.d.'s in cell parameters are only used when they are defined by crystal symmetry. An approximate (isotropic) treatment of cell e.s.d.'s is used for estimating e.s.d.'s involving l.s. planes.
Refinement. Refinement of *F*^2^ against ALL reflections. The weighted *R*-factor *wR* and goodness of fit *S* are based on *F*^2^, conventional *R*-factors *R* are based on *F*, with *F* set to zero for negative *F*^2^. The threshold expression of *F*^2^ > 2σ(*F*^2^) is used only for calculating *R*-factors(gt) *etc*. and is not relevant to the choice of reflections for refinement. *R*-factors based on *F*^2^ are statistically about twice as large as those based on *F*, and *R*- factors based on ALL data will be even larger.

### Fractional atomic coordinates and isotropic or equivalent isotropic displacement parameters (Å^2^)

**Table:**

	*x*	*y*	*z*	*U*_iso_*/*U*_eq_	
Co1	−0.34999 (5)	−0.24482 (4)	0.79340 (3)	0.02013 (13)	
S1	−0.04501 (10)	−0.25459 (8)	0.52827 (6)	0.0281 (2)	
S2	0.25186 (10)	0.02620 (8)	0.60913 (6)	0.0299 (2)	
S3	0.14365 (9)	−0.13442 (8)	0.34898 (6)	0.0240 (2)	
O1	−0.1611 (2)	−0.2315 (2)	0.75483 (15)	0.0264 (6)	
O2	−0.2189 (3)	−0.3579 (3)	0.63494 (19)	0.0516 (9)	
O3	0.2470 (3)	−0.0587 (2)	0.19493 (16)	0.0291 (6)	
O4	0.3956 (2)	0.0885 (2)	0.26610 (16)	0.0257 (5)	
N1	−0.5146 (3)	−0.2245 (3)	0.86423 (19)	0.0248 (6)	
N2	−0.2355 (3)	−0.1859 (2)	0.92703 (18)	0.0238 (6)	
N3	−0.4950 (3)	−0.3311 (2)	0.67959 (19)	0.0227 (6)	
N4	−0.3249 (3)	−0.4266 (3)	0.82227 (19)	0.0249 (6)	
C1	−0.1500 (4)	−0.2735 (3)	0.6790 (2)	0.0279 (8)	
C2	−0.0441 (4)	−0.2122 (3)	0.6389 (2)	0.0239 (7)	
C3	0.0813 (3)	−0.1463 (3)	0.5260 (2)	0.0233 (7)	
C4	0.1506 (3)	−0.1030 (3)	0.4618 (2)	0.0217 (7)	
C5	0.2701 (3)	−0.0221 (3)	0.3501 (2)	0.0223 (7)	
C6	0.3144 (4)	0.0356 (3)	0.4327 (2)	0.0256 (8)	
H6	0.3829	0.0992	0.4458	0.031*	
C7	0.2462 (4)	−0.0108 (3)	0.4965 (2)	0.0247 (8)	
C8	0.1232 (4)	−0.0842 (3)	0.6088 (2)	0.0238 (7)	
C9	0.0504 (4)	−0.1213 (3)	0.6728 (2)	0.0246 (8)	
H9	0.0655	−0.0870	0.7321	0.030*	
C10	0.3060 (4)	0.0040 (3)	0.2626 (2)	0.0222 (7)	
C11	−0.6518 (4)	−0.2416 (3)	0.8323 (3)	0.0340 (9)	
H11	−0.6881	−0.2719	0.7717	0.041*	
C12	−0.7471 (4)	−0.2171 (4)	0.8838 (3)	0.0442 (11)	
H12	−0.8453	−0.2314	0.8588	0.053*	
C13	−0.6955 (4)	−0.1725 (4)	0.9702 (3)	0.0467 (11)	
H13	−0.7585	−0.1541	1.0054	0.056*	
C14	−0.5511 (4)	−0.1535 (4)	1.0079 (3)	0.0367 (10)	
C15	−0.4884 (5)	−0.1077 (4)	1.0981 (3)	0.0436 (11)	
H15	−0.5470	−0.0860	1.1355	0.052*	
C16	−0.3495 (5)	−0.0947 (4)	1.1308 (3)	0.0426 (11)	
H16	−0.3112	−0.0672	1.1920	0.051*	
C17	−0.2559 (4)	−0.1213 (3)	1.0757 (2)	0.0293 (8)	
C18	−0.1105 (4)	−0.1083 (4)	1.1059 (3)	0.0380 (10)	
H18	−0.0664	−0.0832	1.1670	0.046*	
C19	−0.0319 (4)	−0.1316 (4)	1.0472 (3)	0.0466 (11)	
H19	0.0676	−0.1220	1.0669	0.056*	
C20	−0.0977 (4)	−0.1699 (4)	0.9581 (3)	0.0387 (10)	
H20	−0.0411	−0.1851	0.9179	0.046*	
C21	−0.3148 (4)	−0.1624 (3)	0.9851 (2)	0.0227 (7)	
C22	−0.4630 (4)	−0.1814 (3)	0.9514 (2)	0.0260 (8)	
C23	−0.5794 (4)	−0.2829 (3)	0.6092 (2)	0.0286 (8)	
H23	−0.5839	−0.2005	0.6101	0.034*	
C24	−0.6620 (4)	−0.3476 (3)	0.5338 (3)	0.0336 (9)	
H24	−0.7200	−0.3093	0.4845	0.040*	
C25	−0.6588 (4)	−0.4672 (3)	0.5312 (3)	0.0334 (9)	
H25	−0.7151	−0.5124	0.4806	0.040*	
C26	−0.5707 (4)	−0.5212 (3)	0.6049 (2)	0.0280 (8)	
C27	−0.5593 (4)	−0.6450 (3)	0.6079 (3)	0.0334 (9)	
H27	−0.6145	−0.6943	0.5594	0.040*	
C28	−0.4704 (4)	−0.6915 (3)	0.6793 (3)	0.0372 (9)	
H28	−0.4630	−0.7736	0.6798	0.045*	
C29	−0.3870 (4)	−0.6208 (3)	0.7541 (3)	0.0304 (8)	
C30	−0.2932 (5)	−0.6661 (4)	0.8302 (3)	0.0419 (10)	
H30	−0.2806	−0.7474	0.8334	0.050*	
C31	−0.2213 (5)	−0.5919 (4)	0.8988 (3)	0.0447 (11)	
H31	−0.1583	−0.6211	0.9507	0.054*	
C32	−0.2397 (4)	−0.4726 (3)	0.8932 (3)	0.0338 (9)	
H32	−0.1890	−0.4222	0.9424	0.041*	
C33	−0.3986 (3)	−0.5003 (3)	0.7537 (2)	0.0238 (7)	
C34	−0.4911 (3)	−0.4491 (3)	0.6776 (2)	0.0233 (7)	
O5	−0.0843 (5)	−0.5229 (5)	1.1015 (3)	0.1188 (19)	
N5	−0.1221 (5)	−0.4568 (4)	1.2325 (3)	0.0676 (13)	
C35	−0.0501 (6)	−0.5057 (5)	1.1833 (4)	0.0686 (16)	
H35	0.0390	−0.5308	1.2149	0.082*	
C36	−0.0661 (9)	−0.4434 (7)	1.3303 (5)	0.124 (3)	
H36A	0.0319	−0.4643	1.3471	0.186*	
H36B	−0.1212	−0.4946	1.3604	0.186*	
H36C	−0.0712	−0.3622	1.3495	0.186*	
C37	−0.2573 (9)	−0.4182 (8)	1.1872 (7)	0.170 (5)	
H37A	−0.2657	−0.4150	1.1217	0.255*	
H37B	−0.2679	−0.3404	1.2112	0.255*	
H37C	−0.3307	−0.4727	1.1976	0.255*	

### Atomic displacement parameters (Å^2^)

**Table:**

	*U*^11^	*U*^22^	*U*^33^	*U*^12^	*U*^13^	*U*^23^
Co1	0.0232 (2)	0.0210 (2)	0.0178 (2)	−0.00021 (18)	0.00842 (19)	−0.00068 (18)
S1	0.0325 (5)	0.0338 (5)	0.0213 (4)	−0.0060 (4)	0.0147 (4)	−0.0054 (4)
S2	0.0367 (5)	0.0334 (5)	0.0198 (4)	−0.0072 (4)	0.0100 (4)	−0.0036 (4)
S3	0.0295 (5)	0.0259 (5)	0.0189 (4)	−0.0025 (4)	0.0112 (4)	−0.0011 (3)
O1	0.0304 (14)	0.0321 (14)	0.0205 (12)	0.0009 (11)	0.0143 (10)	−0.0038 (10)
O2	0.0568 (19)	0.061 (2)	0.0436 (17)	−0.0321 (16)	0.0342 (15)	−0.0298 (15)
O3	0.0367 (15)	0.0282 (14)	0.0248 (13)	−0.0034 (11)	0.0138 (11)	−0.0016 (11)
O4	0.0277 (13)	0.0236 (13)	0.0267 (13)	−0.0027 (10)	0.0100 (11)	0.0006 (10)
N1	0.0247 (16)	0.0279 (16)	0.0238 (15)	0.0022 (13)	0.0097 (12)	0.0041 (13)
N2	0.0271 (16)	0.0276 (16)	0.0195 (14)	0.0024 (13)	0.0110 (12)	−0.0021 (12)
N3	0.0243 (15)	0.0230 (15)	0.0233 (15)	0.0036 (12)	0.0099 (12)	0.0011 (12)
N4	0.0266 (16)	0.0265 (16)	0.0220 (15)	0.0010 (13)	0.0074 (13)	0.0016 (12)
C1	0.0288 (19)	0.033 (2)	0.0245 (18)	0.0010 (16)	0.0126 (15)	−0.0031 (16)
C2	0.0273 (19)	0.0296 (19)	0.0173 (17)	0.0050 (15)	0.0099 (14)	−0.0002 (14)
C3	0.0249 (18)	0.0282 (19)	0.0182 (17)	0.0029 (15)	0.0080 (14)	−0.0008 (14)
C4	0.0257 (18)	0.0251 (18)	0.0160 (16)	0.0022 (14)	0.0083 (14)	−0.0010 (14)
C5	0.0243 (18)	0.0214 (18)	0.0236 (18)	0.0006 (14)	0.0106 (14)	0.0033 (14)
C6	0.0278 (19)	0.0266 (19)	0.0241 (18)	−0.0002 (15)	0.0102 (15)	0.0007 (15)
C7	0.0301 (19)	0.0272 (19)	0.0171 (17)	0.0013 (15)	0.0068 (15)	0.0013 (14)
C8	0.0265 (18)	0.0259 (19)	0.0209 (17)	0.0024 (15)	0.0094 (14)	0.0006 (14)
C9	0.0304 (19)	0.029 (2)	0.0172 (17)	0.0053 (15)	0.0101 (15)	−0.0002 (14)
C10	0.0259 (18)	0.0206 (18)	0.0224 (17)	0.0062 (14)	0.0096 (14)	0.0022 (14)
C11	0.028 (2)	0.043 (2)	0.032 (2)	0.0019 (17)	0.0105 (17)	0.0071 (18)
C12	0.023 (2)	0.066 (3)	0.050 (3)	0.005 (2)	0.0181 (19)	0.015 (2)
C13	0.041 (3)	0.067 (3)	0.043 (3)	0.014 (2)	0.029 (2)	0.015 (2)
C14	0.038 (2)	0.049 (3)	0.032 (2)	0.0103 (19)	0.0219 (18)	0.0091 (19)
C15	0.050 (3)	0.058 (3)	0.034 (2)	0.013 (2)	0.029 (2)	0.001 (2)
C16	0.061 (3)	0.052 (3)	0.021 (2)	0.007 (2)	0.022 (2)	−0.0047 (19)
C17	0.041 (2)	0.029 (2)	0.0204 (18)	0.0060 (17)	0.0119 (16)	0.0000 (15)
C18	0.044 (2)	0.047 (3)	0.0199 (19)	0.000 (2)	0.0032 (17)	−0.0091 (17)
C19	0.028 (2)	0.069 (3)	0.038 (2)	0.000 (2)	0.0030 (18)	−0.015 (2)
C20	0.026 (2)	0.062 (3)	0.029 (2)	0.0007 (19)	0.0116 (17)	−0.0158 (19)
C21	0.0307 (19)	0.0239 (18)	0.0172 (16)	0.0051 (15)	0.0122 (14)	0.0034 (14)
C22	0.036 (2)	0.0265 (19)	0.0222 (18)	0.0059 (16)	0.0177 (16)	0.0047 (15)
C23	0.027 (2)	0.031 (2)	0.0272 (19)	0.0044 (16)	0.0061 (16)	0.0001 (16)
C24	0.031 (2)	0.041 (2)	0.027 (2)	0.0062 (17)	0.0031 (16)	−0.0017 (17)
C25	0.029 (2)	0.040 (2)	0.028 (2)	0.0009 (17)	0.0030 (16)	−0.0128 (17)
C26	0.0243 (19)	0.030 (2)	0.030 (2)	−0.0011 (15)	0.0083 (16)	−0.0049 (16)
C27	0.033 (2)	0.029 (2)	0.037 (2)	−0.0042 (17)	0.0096 (18)	−0.0117 (17)
C28	0.042 (2)	0.023 (2)	0.047 (3)	0.0001 (17)	0.015 (2)	−0.0041 (18)
C29	0.035 (2)	0.0230 (19)	0.035 (2)	−0.0001 (16)	0.0111 (17)	−0.0020 (16)
C30	0.051 (3)	0.026 (2)	0.046 (3)	0.0064 (19)	0.006 (2)	0.0097 (19)
C31	0.052 (3)	0.034 (2)	0.041 (2)	0.010 (2)	−0.004 (2)	0.0101 (19)
C32	0.039 (2)	0.033 (2)	0.026 (2)	0.0019 (18)	0.0020 (17)	0.0033 (17)
C33	0.0207 (17)	0.0244 (19)	0.0270 (18)	−0.0025 (14)	0.0086 (14)	0.0006 (15)
C34	0.0222 (18)	0.0282 (19)	0.0221 (18)	0.0018 (14)	0.0102 (14)	−0.0005 (15)
O5	0.089 (3)	0.206 (6)	0.052 (3)	−0.016 (3)	0.009 (2)	−0.025 (3)
N5	0.074 (3)	0.068 (3)	0.079 (3)	0.007 (2)	0.051 (3)	0.009 (3)
C35	0.058 (3)	0.099 (5)	0.047 (3)	−0.001 (3)	0.013 (3)	−0.003 (3)
C36	0.173 (8)	0.134 (7)	0.085 (5)	−0.032 (6)	0.080 (5)	−0.041 (5)
C37	0.134 (7)	0.193 (10)	0.247 (11)	0.103 (7)	0.136 (8)	0.151 (9)

### Geometric parameters (Å, °)

**Table:**

Co1—O4^i^	2.047 (2)	C15—C16	1.332 (6)
Co1—O1	2.097 (2)	C15—H15	0.9500
Co1—N3	2.127 (3)	C16—C17	1.440 (5)
Co1—N2	2.130 (3)	C16—H16	0.9500
Co1—N4	2.158 (3)	C17—C18	1.389 (5)
Co1—N1	2.192 (3)	C17—C21	1.411 (5)
S1—C3	1.722 (3)	C18—C19	1.359 (6)
S1—C2	1.740 (3)	C18—H18	0.9500
S2—C7	1.743 (3)	C19—C20	1.393 (5)
S2—C8	1.747 (4)	C19—H19	0.9500
S3—C4	1.730 (3)	C20—H20	0.9500
S3—C5	1.744 (3)	C21—C22	1.424 (5)
O1—C1	1.274 (4)	C23—C24	1.396 (5)
O2—C1	1.233 (4)	C23—H23	0.9500
O3—C10	1.238 (4)	C24—C25	1.376 (5)
O4—C10	1.270 (4)	C24—H24	0.9500
O4—Co1^i^	2.047 (2)	C25—C26	1.407 (5)
N1—C11	1.319 (5)	C25—H25	0.9500
N1—C22	1.363 (4)	C26—C34	1.403 (5)
N2—C20	1.322 (5)	C26—C27	1.434 (5)
N2—C21	1.356 (4)	C27—C28	1.347 (6)
N3—C23	1.327 (4)	C27—H27	0.9500
N3—C34	1.359 (4)	C28—C29	1.428 (5)
N4—C32	1.323 (5)	C28—H28	0.9500
N4—C33	1.356 (4)	C29—C33	1.395 (5)
C1—C2	1.496 (5)	C29—C30	1.411 (5)
C2—C9	1.361 (5)	C30—C31	1.354 (6)
C3—C8	1.390 (4)	C30—H30	0.9500
C3—C4	1.415 (4)	C31—C32	1.394 (5)
C4—C7	1.380 (5)	C31—H31	0.9500
C5—C6	1.363 (5)	C32—H32	0.9500
C5—C10	1.496 (4)	C33—C34	1.435 (5)
C6—C7	1.415 (5)	O5—C35	1.212 (6)
C6—H6	0.9500	N5—C35	1.297 (6)
C8—C9	1.412 (4)	N5—C37	1.434 (9)
C9—H9	0.9500	N5—C36	1.448 (8)
C11—C12	1.409 (5)	C35—H35	0.9500
C11—H11	0.9500	C36—H36A	0.9800
C12—C13	1.358 (6)	C36—H36B	0.9800
C12—H12	0.9500	C36—H36C	0.9800
C13—C14	1.397 (6)	C37—H37A	0.9800
C13—H13	0.9500	C37—H37B	0.9800
C14—C22	1.417 (5)	C37—H37C	0.9800
C14—C15	1.427 (6)		
			
O4^i^—Co1—O1	87.55 (9)	C14—C15—H15	119.3
O4^i^—Co1—N3	89.33 (10)	C15—C16—C17	121.6 (4)
O1—Co1—N3	104.10 (10)	C15—C16—H16	119.2
O4^i^—Co1—N2	100.37 (10)	C17—C16—H16	119.2
O1—Co1—N2	88.27 (10)	C18—C17—C21	117.5 (3)
N3—Co1—N2	164.68 (11)	C18—C17—C16	124.2 (4)
O4^i^—Co1—N4	165.78 (10)	C21—C17—C16	118.3 (4)
O1—Co1—N4	91.40 (10)	C19—C18—C17	119.5 (4)
N3—Co1—N4	77.16 (11)	C19—C18—H18	120.3
N2—Co1—N4	93.77 (11)	C17—C18—H18	120.3
O4^i^—Co1—N1	89.40 (10)	C18—C19—C20	119.7 (4)
O1—Co1—N1	163.91 (10)	C18—C19—H19	120.1
N3—Co1—N1	91.65 (11)	C20—C19—H19	120.1
N2—Co1—N1	76.75 (11)	N2—C20—C19	122.9 (3)
N4—Co1—N1	95.35 (11)	N2—C20—H20	118.5
C3—S1—C2	91.21 (16)	C19—C20—H20	118.5
C7—S2—C8	90.49 (16)	N2—C21—C17	122.7 (3)
C4—S3—C5	90.95 (16)	N2—C21—C22	117.4 (3)
C1—O1—Co1	122.3 (2)	C17—C21—C22	120.0 (3)
C10—O4—Co1^i^	124.4 (2)	N1—C22—C14	122.5 (3)
C11—N1—C22	118.2 (3)	N1—C22—C21	117.8 (3)
C11—N1—Co1	129.0 (2)	C14—C22—C21	119.7 (3)
C22—N1—Co1	112.7 (2)	N3—C23—C24	123.1 (4)
C20—N2—C21	117.7 (3)	N3—C23—H23	118.5
C20—N2—Co1	127.0 (2)	C24—C23—H23	118.5
C21—N2—Co1	115.3 (2)	C25—C24—C23	119.7 (4)
C23—N3—C34	117.7 (3)	C25—C24—H24	120.2
C23—N3—Co1	127.6 (2)	C23—C24—H24	120.2
C34—N3—Co1	114.5 (2)	C24—C25—C26	118.8 (3)
C32—N4—C33	117.7 (3)	C24—C25—H25	120.6
C32—N4—Co1	128.3 (3)	C26—C25—H25	120.6
C33—N4—Co1	113.7 (2)	C34—C26—C25	117.6 (3)
O2—C1—O1	126.8 (3)	C34—C26—C27	119.7 (3)
O2—C1—C2	116.9 (3)	C25—C26—C27	122.8 (3)
O1—C1—C2	116.3 (3)	C28—C27—C26	120.1 (4)
C9—C2—C1	130.4 (3)	C28—C27—H27	120.0
C9—C2—S1	112.5 (2)	C26—C27—H27	120.0
C1—C2—S1	116.9 (2)	C27—C28—C29	121.8 (4)
C8—C3—C4	112.3 (3)	C27—C28—H28	119.1
C8—C3—S1	110.8 (2)	C29—C28—H28	119.1
C4—C3—S1	136.9 (3)	C33—C29—C30	117.3 (3)
C7—C4—C3	112.8 (3)	C33—C29—C28	119.2 (3)
C7—C4—S3	111.1 (2)	C30—C29—C28	123.6 (4)
C3—C4—S3	136.1 (3)	C31—C30—C29	119.0 (4)
C6—C5—C10	129.3 (3)	C31—C30—H30	120.5
C6—C5—S3	112.4 (2)	C29—C30—H30	120.5
C10—C5—S3	118.1 (2)	C30—C31—C32	120.0 (4)
C5—C6—C7	111.8 (3)	C30—C31—H31	120.0
C5—C6—H6	124.1	C32—C31—H31	120.0
C7—C6—H6	124.1	N4—C32—C31	122.7 (4)
C4—C7—C6	113.7 (3)	N4—C32—H32	118.6
C4—C7—S2	112.3 (2)	C31—C32—H32	118.6
C6—C7—S2	134.0 (3)	N4—C33—C29	123.2 (3)
C3—C8—C9	113.7 (3)	N4—C33—C34	117.0 (3)
C3—C8—S2	112.1 (2)	C29—C33—C34	119.8 (3)
C9—C8—S2	134.2 (3)	N3—C34—C26	123.3 (3)
C2—C9—C8	111.7 (3)	N3—C34—C33	117.2 (3)
C2—C9—H9	124.1	C26—C34—C33	119.5 (3)
C8—C9—H9	124.1	C35—N5—C37	118.1 (6)
O3—C10—O4	126.7 (3)	C35—N5—C36	120.1 (6)
O3—C10—C5	117.4 (3)	C37—N5—C36	121.8 (6)
O4—C10—C5	115.9 (3)	O5—C35—N5	127.0 (6)
N1—C11—C12	123.1 (4)	O5—C35—H35	116.5
N1—C11—H11	118.4	N5—C35—H35	116.5
C12—C11—H11	118.4	N5—C36—H36A	109.5
C13—C12—C11	118.5 (4)	N5—C36—H36B	109.5
C13—C12—H12	120.7	H36A—C36—H36B	109.5
C11—C12—H12	120.7	N5—C36—H36C	109.5
C12—C13—C14	120.9 (4)	H36A—C36—H36C	109.5
C12—C13—H13	119.6	H36B—C36—H36C	109.5
C14—C13—H13	119.6	N5—C37—H37A	109.5
C13—C14—C22	116.7 (4)	N5—C37—H37B	109.5
C13—C14—C15	124.4 (4)	H37A—C37—H37B	109.5
C22—C14—C15	118.9 (4)	N5—C37—H37C	109.5
C16—C15—C14	121.4 (4)	H37A—C37—H37C	109.5
C16—C15—H15	119.3	H37B—C37—H37C	109.5
			
O4^i^—Co1—O1—C1	−90.8 (3)	Co1^i^—O4—C10—O3	70.5 (4)
N3—Co1—O1—C1	−2.1 (3)	Co1^i^—O4—C10—C5	−110.2 (3)
N2—Co1—O1—C1	168.8 (3)	C6—C5—C10—O3	−176.8 (3)
N4—Co1—O1—C1	75.0 (3)	S3—C5—C10—O3	−2.5 (4)
N1—Co1—O1—C1	−170.0 (4)	C6—C5—C10—O4	3.7 (5)
O4^i^—Co1—N1—C11	77.8 (3)	S3—C5—C10—O4	178.1 (2)
O1—Co1—N1—C11	156.8 (4)	C22—N1—C11—C12	0.2 (6)
N3—Co1—N1—C11	−11.5 (3)	Co1—N1—C11—C12	−175.2 (3)
N2—Co1—N1—C11	178.6 (3)	N1—C11—C12—C13	0.7 (6)
N4—Co1—N1—C11	−88.8 (3)	C11—C12—C13—C14	−1.3 (7)
O4^i^—Co1—N1—C22	−97.9 (2)	C12—C13—C14—C22	0.9 (6)
O1—Co1—N1—C22	−18.9 (5)	C12—C13—C14—C15	−179.7 (4)
N3—Co1—N1—C22	172.8 (2)	C13—C14—C15—C16	178.1 (4)
N2—Co1—N1—C22	2.9 (2)	C22—C14—C15—C16	−2.4 (6)
N4—Co1—N1—C22	95.5 (2)	C14—C15—C16—C17	2.8 (7)
O4^i^—Co1—N2—C20	−94.9 (3)	C15—C16—C17—C18	179.3 (4)
O1—Co1—N2—C20	−7.7 (3)	C15—C16—C17—C21	−0.2 (6)
N3—Co1—N2—C20	136.6 (4)	C21—C17—C18—C19	2.1 (6)
N4—Co1—N2—C20	83.6 (3)	C16—C17—C18—C19	−177.5 (4)
N1—Co1—N2—C20	178.2 (4)	C17—C18—C19—C20	−0.9 (7)
O4^i^—Co1—N2—C21	84.7 (2)	C21—N2—C20—C19	0.8 (6)
O1—Co1—N2—C21	171.9 (2)	Co1—N2—C20—C19	−179.6 (3)
N3—Co1—N2—C21	−43.8 (5)	C18—C19—C20—N2	−0.6 (7)
N4—Co1—N2—C21	−96.8 (2)	C20—N2—C21—C17	0.5 (5)
N1—Co1—N2—C21	−2.2 (2)	Co1—N2—C21—C17	−179.2 (3)
O4^i^—Co1—N3—C23	−4.7 (3)	C20—N2—C21—C22	−179.2 (3)
O1—Co1—N3—C23	−92.0 (3)	Co1—N2—C21—C22	1.1 (4)
N2—Co1—N3—C23	124.9 (4)	C18—C17—C21—N2	−1.9 (5)
N4—Co1—N3—C23	179.7 (3)	C16—C17—C21—N2	177.7 (3)
N1—Co1—N3—C23	84.6 (3)	C18—C17—C21—C22	177.8 (3)
O4^i^—Co1—N3—C34	170.2 (2)	C16—C17—C21—C22	−2.7 (5)
O1—Co1—N3—C34	82.9 (2)	C11—N1—C22—C14	−0.7 (5)
N2—Co1—N3—C34	−60.1 (5)	Co1—N1—C22—C14	175.5 (3)
N4—Co1—N3—C34	−5.3 (2)	C11—N1—C22—C21	−179.5 (3)
N1—Co1—N3—C34	−100.4 (2)	Co1—N1—C22—C21	−3.4 (4)
O4^i^—Co1—N4—C32	161.2 (4)	C13—C14—C22—N1	0.1 (6)
O1—Co1—N4—C32	75.6 (3)	C15—C14—C22—N1	−179.4 (3)
N3—Co1—N4—C32	179.7 (3)	C13—C14—C22—C21	179.0 (4)
N2—Co1—N4—C32	−12.8 (3)	C15—C14—C22—C21	−0.5 (6)
N1—Co1—N4—C32	−89.8 (3)	N2—C21—C22—N1	1.6 (5)
O4^i^—Co1—N4—C33	−12.6 (5)	C17—C21—C22—N1	−178.1 (3)
O1—Co1—N4—C33	−98.2 (2)	N2—C21—C22—C14	−177.3 (3)
N3—Co1—N4—C33	5.9 (2)	C17—C21—C22—C14	3.0 (5)
N2—Co1—N4—C33	173.4 (2)	C34—N3—C23—C24	−0.7 (5)
N1—Co1—N4—C33	96.4 (2)	Co1—N3—C23—C24	174.1 (3)
Co1—O1—C1—O2	−28.4 (5)	N3—C23—C24—C25	0.8 (6)
Co1—O1—C1—C2	150.4 (2)	C23—C24—C25—C26	−0.6 (5)
O2—C1—C2—C9	−176.7 (4)	C24—C25—C26—C34	0.4 (5)
O1—C1—C2—C9	4.4 (6)	C24—C25—C26—C27	−179.3 (3)
O2—C1—C2—S1	8.0 (5)	C34—C26—C27—C28	−1.4 (5)
O1—C1—C2—S1	−170.9 (3)	C25—C26—C27—C28	178.3 (4)
C3—S1—C2—C9	−1.2 (3)	C26—C27—C28—C29	0.9 (6)
C3—S1—C2—C1	174.9 (3)	C27—C28—C29—C33	0.5 (6)
C2—S1—C3—C8	1.8 (3)	C27—C28—C29—C30	179.7 (4)
C2—S1—C3—C4	−176.5 (4)	C33—C29—C30—C31	0.8 (6)
C8—C3—C4—C7	1.4 (4)	C28—C29—C30—C31	−178.4 (4)
S1—C3—C4—C7	179.6 (3)	C29—C30—C31—C32	−0.5 (7)
C8—C3—C4—S3	−177.0 (3)	C33—N4—C32—C31	1.4 (6)
S1—C3—C4—S3	1.2 (6)	Co1—N4—C32—C31	−172.2 (3)
C5—S3—C4—C7	−0.1 (3)	C30—C31—C32—N4	−0.7 (7)
C5—S3—C4—C3	178.4 (4)	C32—N4—C33—C29	−1.0 (5)
C4—S3—C5—C6	0.0 (3)	Co1—N4—C33—C29	173.5 (3)
C4—S3—C5—C10	−175.2 (3)	C32—N4—C33—C34	179.6 (3)
C10—C5—C6—C7	174.6 (3)	Co1—N4—C33—C34	−5.9 (4)
S3—C5—C6—C7	0.0 (4)	C30—C29—C33—N4	−0.1 (5)
C3—C4—C7—C6	−178.7 (3)	C28—C29—C33—N4	179.2 (3)
S3—C4—C7—C6	0.1 (4)	C30—C29—C33—C34	179.3 (3)
C3—C4—C7—S2	−0.7 (4)	C28—C29—C33—C34	−1.5 (5)
S3—C4—C7—S2	178.14 (18)	C23—N3—C34—C26	0.5 (5)
C5—C6—C7—C4	−0.1 (5)	Co1—N3—C34—C26	−175.0 (3)
C5—C6—C7—S2	−177.5 (3)	C23—N3—C34—C33	179.5 (3)
C8—S2—C7—C4	−0.1 (3)	Co1—N3—C34—C33	4.0 (4)
C8—S2—C7—C6	177.4 (4)	C25—C26—C34—N3	−0.3 (5)
C4—C3—C8—C9	176.7 (3)	C27—C26—C34—N3	179.4 (3)
S1—C3—C8—C9	−2.0 (4)	C25—C26—C34—C33	−179.3 (3)
C4—C3—C8—S2	−1.5 (4)	C27—C26—C34—C33	0.4 (5)
S1—C3—C8—S2	179.82 (18)	N4—C33—C34—N3	1.3 (4)
C7—S2—C8—C3	0.9 (3)	C29—C33—C34—N3	−178.0 (3)
C7—S2—C8—C9	−176.8 (4)	N4—C33—C34—C26	−179.6 (3)
C1—C2—C9—C8	−175.2 (4)	C29—C33—C34—C26	1.0 (5)
S1—C2—C9—C8	0.3 (4)	C37—N5—C35—O5	0.1 (10)
C3—C8—C9—C2	1.1 (4)	C36—N5—C35—O5	179.7 (6)
S2—C8—C9—C2	178.8 (3)		

Symmetry codes: (i) −*x*, −*y*, −*z*+1.
